# Higher Fibroblast Growth Factor 23 Levels Are Causally Associated With Lower Bone Mineral Density of Heel and Femoral Neck: Evidence From Two-Sample Mendelian Randomization Analysis

**DOI:** 10.3389/fpubh.2020.00467

**Published:** 2020-09-02

**Authors:** Yue Wang, Hui Wang, Peizhan Chen

**Affiliations:** ^1^Center for Single-Cell Omics, School of Public Health, Shanghai Jiao Tong University School of Medicine, Shanghai, China; ^2^Clinical Research Center, Ruijin Hospital North, Shanghai Jiao Tong University School of Medicine, Shanghai, China

**Keywords:** fibroblast growth factor 23 (FGF23), single nucleotide polymorphisms (SNPs), heel, femoral neck, bone mineral density, mendelian randomization

## Abstract

**Background:** Previous observational studies have indicated that high levels of fibroblast growth factor 23 (FGF23), a phosphoric hormone that inhibits calcitriol synthesis, in the blood is associated with the reduced bone mineral density (BMD); however, whether this association is causal remains unclear. In this study, we conducted a Mendelian Random (MR) study to investigate whether the genetic predisposition of higher FGF23 levels was causally associated with lower BMD in adults.

**Methods:** A two-sample MR was performed with five single nucleotide polymorphisms significantly associated with FGF23, selected as instrumental variables. Two-sample MR estimates were derived from summary-level data of large-sample genome-wide association studies for BMD and the levels of bone metabolism characteristics.

**Results:** The two-sample MR analysis showed that for every 1-unit increase in the log-transformed blood FGF23 level (pg/mL), the decreased levels of adult heel BMD (β = −0.201, se = 0.084, *P* = 0.016) and femoral neck BMD (β = −0.286, se = 0.126, *P* = 0.022) were noted, indicative of a causal relationship based on the inverse variance weighting method. However, FGF23 levels were not correlated with adult lumbar spine BMD (β = −0.166, se = 0.193, *P* = 0.389), and forearm BMD (β = −0.186, se = 0.366, *P* = 0.610). Moreover, the two-sample MR analysis suggested that there was no evidence for associations between FGF23 and adult calcium, phosphorus, 25(OH)D, creatinine, and magnesium levels.

**Conclusions:** This study suggests that there may be a causal relationship between blood FGF23 levels and BMD of the heel and femoral neck in adults; however, more investigations are necessary to determine whether FGF23 may be a potential biomarker and/or therapeutic target for diseases that affect bone mineralization.

## Introduction

Fibroblast growth factor 23 (FGF23), a new type of phosphorylation biomarker, is a member of the FGF polypeptide family. FGF23 has multiple functions in regulating mineral metabolism and development. Primarily produced by osteoblasts and bone cells, FGF23 can regulate phosphate homeostasis and 1α,25(OH)_2_-D metabolism via a specific FGF receptor-α-klotho-complex in renal tubular kidney cells, thereby participating in the regulation of bone and kidney axis (1–3). FGF23 is synthesized by osteoblasts and osteoclasts and is involved in the regulation of mineral metabolism. It can promote phosphate excretion by inhibiting sodium phosphate cotransporter expression in renal proximal tubular cells, thus, high FGF23 levels would lead to hypophosphatemia (3). Higher FGF23 levels could also lead to mineralization damage and a lack of ossification in cartilage (1–3). Moreover, FGF23 can also inhibit calcitriol levels by stimulating 1,24,25-hydroxylase (CYP24A1) mediated calcitriol degradation and inhibiting the 1,25α-hydroxylase (CYP27B1) mediated calcitriol synthesis (4, 5). FGF23 is also associated with the serum phosphate through modulating the NaPi-2a protein level in brush border membrane of renal proximal tubules (5). When FGF23 is deficient, renal reabsorption of phosphate and 1α,25-dihydroxy vitamin D [1α,25(OH)_2_D] synthesis increases; the serum phosphate and calcium levels change accordingly (6–9). In mice models, FGF23 leads to growth retardation, osteomalacia, and destroys phosphate homeostasis. Overexpression of FGF23 can also inhibit osteoblast differentiation and cause matrix mineralization in cells (10, 11).

Bone mineral density (BMD) is clinically employed as the predictive indicator of osteoporosis and fracture risk. Studies have determined the associations between FGF23 levels and BMD; however, results were not continually consistent. A large retrospective cohort study of the elderly population has suggested that a high level of FGF23 was associated with a decrease in BMD of the hip (12). Bouksila et al. showed that the level of FGF23 was negatively correlated with the BMD in the lumbar spine (LS) (13). However, results from some other epidemiological studies did not support the association between FGF23 levels and BMD in lumbar spine (14, 15). Typically, existing epidemiological studies focused on observational studies and they are difficulty to conclude the causality using standard statistical instruments. Although previous observational studies sought to increase their credibility by adjusting confounding factors, it was almost impossible to control all confounding factors. Thus, evidence of the correlation between FGF23 and BMD may be influenced by unadjusted confounding factors.

As an advanced statistical method, the Mendelian Randomization (MR) study can help establish the causal relationship between target exposures (such as FGF23 in this study) and outcomes using single nucleotide polymorphisms (SNPs) as instrumental variables (IVs) (16–21). As genetic randomization occurs prior to the onset of disease, MR analysis also reduces reverse causality. Thus, MR analysis has been widely used to assess the causal relationship between modifiable factors and disease onsets (22). Considering that the biological effects of hematuria and 1α,25(OH)2D deficiency are associated with higher FGF23 levels, we hypothesized that higher blood FGF23 concentrations were causally associated with lower BMD in adults. Recently, SNPs significantly associated with blood FGF23 levels have been identified through a large-scale genome-wide association study (GWAS), making it possible to infer the causality relationship of genetic predisposition blood FGF23 levels and BMD with MR studies (23). Herein, we conducted a two-sample MR analysis based on summary statistics of a large-scale GWAS to explore the causal relationships between FGF23 levels and BMD.

## Materials and Methods

### Study Design

**Figure 1:**
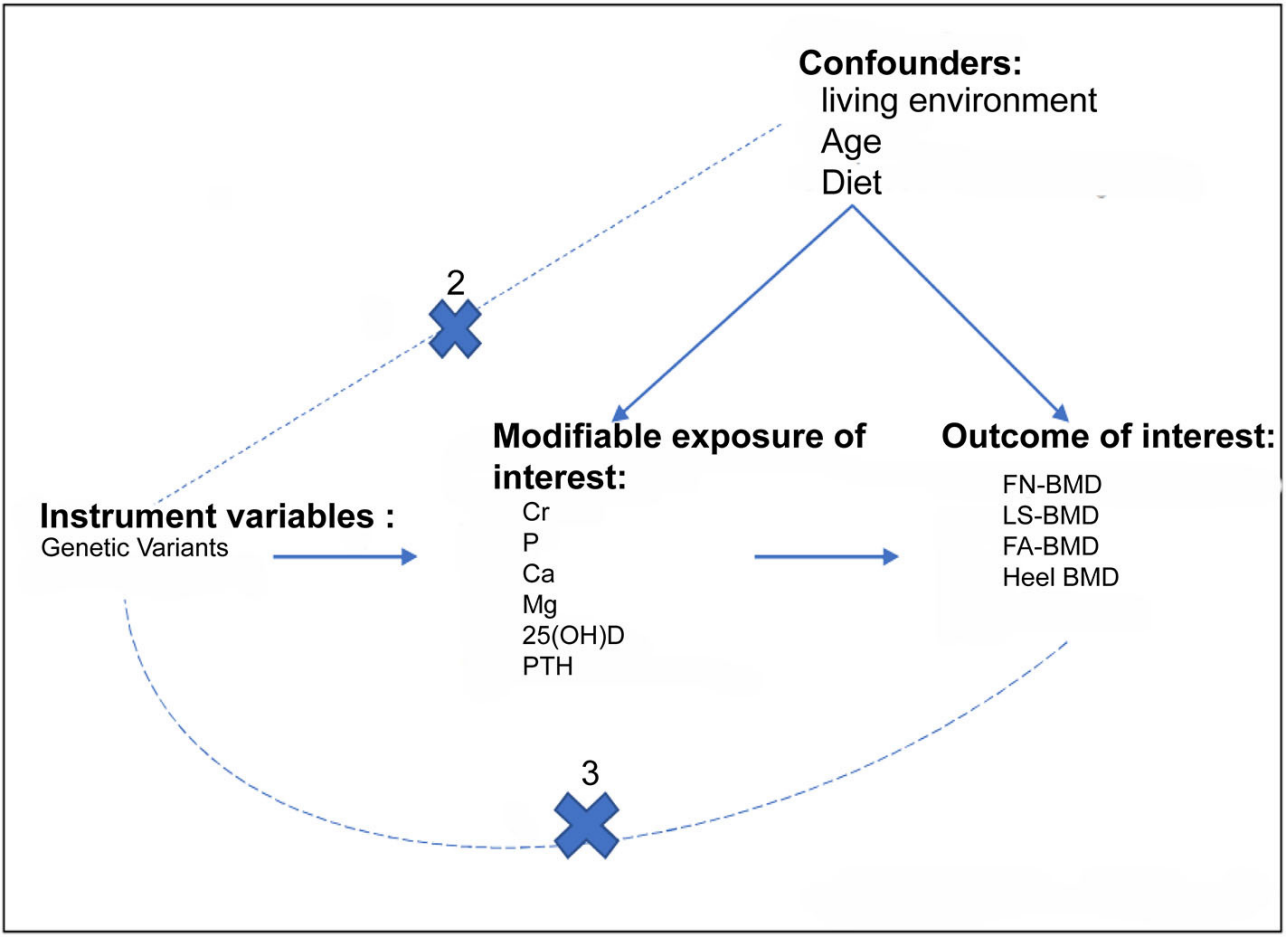
Assumptions for the two-sample Mendelian Randomization and study design. The three assumptions include: (1) the genetic variants should be strongly correlated with exposure; (2) the genetic variants should not be correlated with exposure-results confounding factors; (3) the genetic variants must only be through exposure and not through any other pathways to influence the results. The dotted line indicates an approach to violate the assumption. The primary outcomes of the study were adult BMD, whereas the secondary outcomes were Cr, P, Ca, Mg, and VD. Cr, serum creatinine; P, Serum phosphorus; Ca, Calcium; Mg, Magnesium.

To determine the causal relationships between FGF23 levels and BMD in adults, we conducted a two-sample MR study based on public summary-level data derived from the GWAS (Figure 1). The focus of this study was on BMD values in four different bone sites: femoral neck mineral density (FN-BMD), lumbar spine BMD (LS-BMD), forearm BMD (FA-BMD), and heel bone mineral density (heel-BMD). Moreover, we evaluated the contribution of FGF23 to bone metabolism related traits and osteoarthritis (secondary results; Figure 1).

### Instrumental Variable Selection and Validation

Prior to conducting the two-sample MR study, it should be determined whether genetic IVs satisfied the following three assumptions: (1) genetic variation should be associated with FGF23 levels; (2) genetic variation must not be related to any confounding factors; (3) there is no direct correlation between genetic variation and the outcome, or any way other than the exposure to correlate with the outcome. For assumption 1, five single nucleotide polymorphisms (SNPs) that reached genome-wide significance at the level of *P* < 5 × 10^−8^ during the GWAS were included, and these loci accounted for about 3% of FGF23 variation (23). For assumption 2, based on European ancestry, we used cluster functions implemented in the TwoSampleMR package of R (www.r-project.org) to assess linkage disequilibrium between loci (LD). These SNPs were suitable as IV for MR studies as the correlation coefficient between different SNPs was negligible (*r*^2^ < 0.01; Table 1).

**Table 1:** Characteristics of five SNP loci included in the Mendelian randomization (MR) analysis.

**SNP^**a**^**	**Chr**	**Position**	**Nearest gene^**b**^**	**FGF23 increasing allele**	**Other allele**	**FGF23 increasing allele frequency^**c**^**	**β^**d**^**	**se**	***P-*value**	***n***
rs17216707^a^	20	52,732,362	CYP24A1	T	C	0.8	0.054	0.005	3.0 × 10^−24^	16,624
rs2769071	9	136,145,974	ABO	G	A	0.37	0.037	0.005	6.1 × 10^−17^	16,624
rs11741640	5	176,792,743	RGS14	G	A	0.73	0.039	0.005	1.6 × 10^−16^	16,624
rs17479566	9	71,198,013	LINC01506	T	C	0.22	0.031	0.005	2.0 × 10^−9^	16,624
rs9925837	16	79,927,303	LINC01229	G	A	0.13	0.035	0.006	5.1 × 10^−9^	16,624

a *rs17216707 cannot be obtained in the results (FN-, LS- and FA-BMD) study, and the proxy SNP for the relevant SNP at high LD (r^2^ > 0.8) was not identified*.

b *Nearest gene by physical distance to the lead SNP*.

c *Allele frequency data from 1,000 Genomes Phase 1 genotype data*.

d *β-estimates are interpreted as the relative difference in FGF23 concentration per minor allele; e.g., 0.051 is a 5.1% higher FGF23 concentration per additional allele*.

### Data Sources

Summary statistics for FGF23-related SNPs (β-coefficients and standard errors) were extracted from a meta-analysis GWAS conducted by the ReproGen Alliance, which is composed of 16,624 European ancestries from seven cohort studies (23). Detailed information is provided in Table 1 and Supplementary Table 1. Using European individual summary statistics of FN-, LS-, and FA-BMD from the GEnetic Factors for OSteoporosis (GEFOS) alliance, impact estimates of these FGF23-related SNPs on OP risk were evaluated (24). As the target SNP (rs17216707) cannot be obtained in the results (FN-, LS-, and FA-BMD) study, and the proxy SNP for the relevant SNP at high LD (*r*^2^ > 0.8) was not identified, only the remaining four SNPs (rs11741640, rs9925837, rs2769071, and rs17479566) were applied as IVs for FGF23 in assessing FN-, LS-, and FA-BMD.

Summary-level data (β-coefficients and standard errors) for heel BMD and serum bone metabolism-related traits were obtained from the public phenome-wide association studies (PheWAS) database (25). Summary statistics from these consortia were retrieved from the following public websites: http://phewas.mrbase.org/, GEFOS, http://www.gefos.org/?q=content/data-release-2015. For bone metabolism traits, summary-level data were obtained from meta-analysis of the MRC Integrative Epidemiology Unit (MRC-IEU) catalog, including serum calcium (*n* = 64,979), serum phosphorus (*n* = 463,010), serum total vitamin D (*n* = 64,979), serum magnesium (*n* = 64,979), and creatinine (*n* = 24,810).

### Pleiotropy Assessment

An important prerequisite for the MR method is that exposure-related SNPs affect FN-, LS-, FA-, and heel-BMD only through exposure itself (FGF23). To assess whether IVs affect the level of pleiotropic effects of BMD through more than one biological pathway (26), we used the MR-Egger regression to test for evidence of pleiotropy (27, 28). Intercepts that deviate from the origin may provide evidence for potential multiple pleiotropy effects in genetic IVs.

### Statistical Analysis

To evaluate the causal effects of FGF23 on BMD and serum bone metabolism characteristics in adults, we conducted a two-Sample MR study using three different models, including: (1) the conventional inverse-variance weighted (IVW) model; (2) the weighted median model; and (3) the MR-Egger regression model. The weighted median model could provide a consistent estimate of causality when at least 50% of genetic IVs is effective. The MR-Egger regression model can also evaluate directional pleiotropy of IVs (27, 28). The advantage of the MR-Egger regression model is that it evaluates the null causal hypothesis under the assumption of Instrument Strength Independent of Direct Effect (InSIDE). Even if all SNPs included in the selection are invalid, MR-Egger can still provide a robust unbiased estimate (29). The heterogeneity between the causal results estimated by SNPs was calculated under the conventional IVW model, and the leave-one-out sensitivity method was performed to compute whether random estimates were affected by an individual genetic locus (30). MR analysis was performed using TwoSampleMR and MendelianRandomization software packages (31, 32). All statistical tests were two-sided and the statistical significance was set at the level of *P* < 0.05.

## Results

### Causal Effects of FGF23 Levels on BMD Values in Different Sites

**Figure 2:**
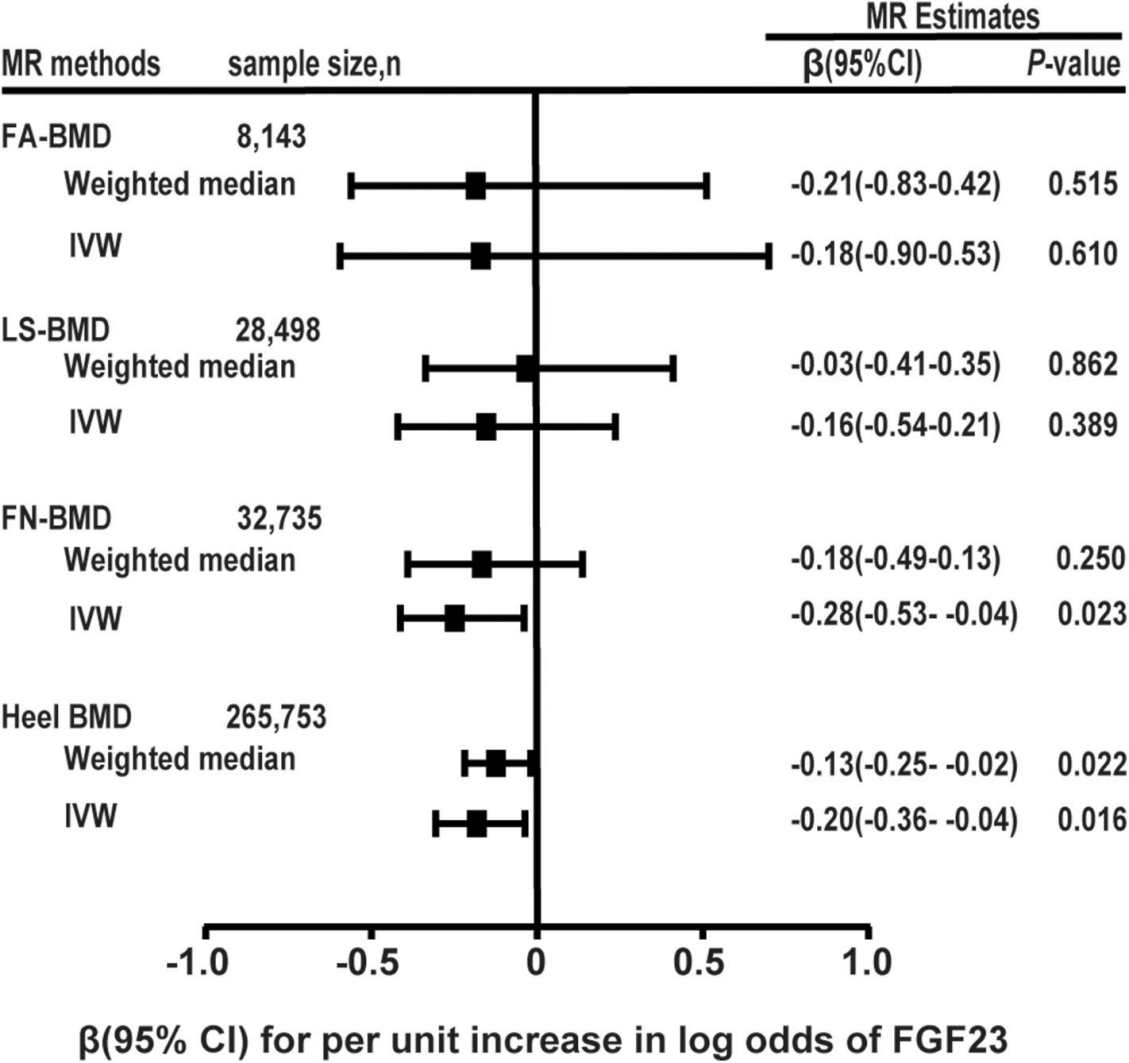
Two-sample MR analysis results of FGF23 and the risks of BMD using the conventional inverse variance weighted (IVW) and weighted median methods. Results are standardized to a logarithmic unit increase in FGF23. FN-BMD, femoral neck BMD, LS-BMD, lumbar spine BMD, FA-BMD, forearm BMD.

In the MR analysis based on the IVW model of five effective SNPs significantly associated with FGF23 (Table 1), we found that a logarithmic unit increment of the blood FGF23 (pg/mL) level was associated with a 0.201-SD lower of heel BMD in the IVW model (β = −0.201, se = 0.084, *P* = 0.016). Similar results were obtained for the weighted median method (Table 2 and Figure 2). There was significant heterogeneity for causal estimates of different SNPs (*P*-heterogeneity = 0.003) in the conventional IVW model. The MR-Egger regression analysis suggested that SNPs violated the multiple effect hypothesis (intercept = 0.045, se = 0.009; *P* < 0.001; Table 2). This indicates that it is versatile, and after correction, we found that the multi-effect adjusted coefficient also supports the causal relationship between FGF23 levels and the decrease in adult BMD (β = −1.152, se = 0.224; *P* < 0.001).

**Table 2:** Two-sample MR analyses of FGF23 and BMD levels.

			**MR estimates, per unit increase in log transformed FGF23 level**					**MR-egger regression**			
			**IVW**			**Weighted median**		**MR-egger**		**Intercept**	
**Outcomes**	**SNPs n**	**Data source, sample size**	**β (se)**	***P-*value**	***P-*heterogeneity**	**β (se)**	***P-*value**	**β (se)**	***P-*value**	**β (se)**	***P-*value**
FA-BMD	4	GEFOS 8,143	−0.186 (0.365)	0.610	0.104	−0.205 (0.321)	0.522	−0.819 (5.442)	0.880	0.036 (0.193)	0.850
LS-BMD	4	GEFOS 28,498	−0.166 (0.193)	0.389	0.141	−0.033 (0.194)	0.863	−3.030 (1.648)	0.060	0.116 (0.059)	0.049
FN-BMD	4	GEFOS 32,735	−0.285 (0.126)	0.022	0.392	−0.182 (0.160)	0.254	−3.939 (1.537)	0.010	−0.016 (0.015)	0.270
Heel-BMDD BMD	5	MRC-IEU 265,753	−0.201 (0.084)	0.016	0.003	−0.132 (0.057)	0.021	−1.152 (0.224)	<0.001	0.045 (0.009)4)	<0.001

*FN-BMD, femoral neck mineral density BMD, LS-BMD, lumbar spine BMD; FA-BMD, forearm BMD; MRC-IEU, Methodology Research Grant-the Integrative Epidemiology Unit (http://www.ewascatalog.org/about/)*.

For each logarithmic unit increase of FGF23 (pg/mL) level, a 0.286-SD lower (β = −0.286, se = 0.126, *P* = 0.022; Table 2 and Figure 2) of adult FN-BMD was observed for the IVW model. The MR-Egger model also suggested that a higher FGF23 level was associated with the reduction of FN BMD in adults (β = −3.939, se = 1.537; *P* = 0.010). The weighted median model also suggested the same result as the MR-Egger model; however, the result was not statistically significant.

The two-sample MR analysis based on the IVW model with four effective IVs associated with FGF23 suggested that there was no significant association between FGF23 level and the FA-BMD (β = −0.186, se = 0.366, *P* = 0.610) or LS-BMD (β = −0.166, se = 0.193, *P* = 0.389; Table 2 and Figure 2). The weighted median and MR-Egger models provided similar results (Figure 2 and Table 2).

### Causal Effects of FGF23 on Adult Serum Bone Metabolic Related Traits

Using the conventional IVW model (Table 1), we found that there was no obvious causal relationship between the FGF23 level and serum bone metabolic related traits, including serum calcium (β = 0.066, se = 0.073; *P* = 0.366), serum phosphorus (β = 0.000, se = 0.001; *P* = 0.866), 25(OH)D (β = −0.08, se = 0.075; *P* = 0.284), creatinine (β = 0.015, se = 0.123; *P* = 0.898), and magnesium (β = 0.013, se = 0.087; *P* = 0.874) (Table 3). For calcium, phosphorus, total vitamin D, magnesium, and creatinine, the heterogeneity between causal estimates among SNPs was not significant (Table 3). Similar results were obtained for the weighted median and MR-Egger models.

**Table 3:** Two-sample MR analyses of FGF23 and related bone metabolism trait levels.

			**MR estimates, per unit increase in log odds of having FGF23**					**MR-Egger regression**			
			**IVW**			**Weighted median**		**Intercept**		**MR-Egger**	
**Outcomes**	**SNPs n**	**Data Source, sample size**	**β (se)**	***P-*value**	***P-*heterogeneity**	**β (se)**	***P-*value**	**β (se)**	***P-*value**	**β (se)**	***P-*value**
Ca	5	MRC-IEU 64,979	0.066 (0.073)	0.366	0.609	0.035 (0.088)	0.689	−0.005 (0.014)	0.709	0.050 (0.348)	0.886
P	5	MRC-IEU 463,010	<0.001 (<0.001)	0.866	0.174	<0.001 (<0.001)	0.650	0.000 (0.000)	0.220	−0.001 (0.001)	0.385
VD	5	MRC-IEU 64,979	−0.080 (0.075)	0.284	0.728	−0.095 (0.089)	0.289	−0.016 (0.015)	0.270	0.402 (0.353)	0.254
Cr	5	CKDGen 24,810	0.015 (0.123)	0.898	0.026	0.027 (0.074)	0.715	−0.144 (0.424)	0.735	4.390 (10.885)	0.687
Mg	5	MRC-IEU 64,979	0.013 (0.087)	0.874	0.217	0.049 (0.099)	0.616	−0.016 (0.016)	0.310	0.445 (0.381)	0.243
OA	5	MRC-IEU 501,405 (case, 38,472/control, 462,933)	−0.001 (0.009)	0.944	0.218	0.001 (2.874)	0.941	0.000 (0.002)	0.979	0.011 (0.037)	0.759

*Cr, serum creatinine; P, serum phosphorus; Ca, Calcium; Mg, Magnesium; OA, Osteoarthritis; VD, Vitamin D; SNP, single nucleotide polymorphism; MR, Mendelian Randomization; SD, standard deviation; CKDGen, CKDGen Consortium; MRC-IEU, The Medical Research Council Integrative Epidemiology Unit (http://www.bristol.ac.uk/integrative-epidemiology/)*.

### Sensitivity Analyses of MR Studies

**Figure 3:**
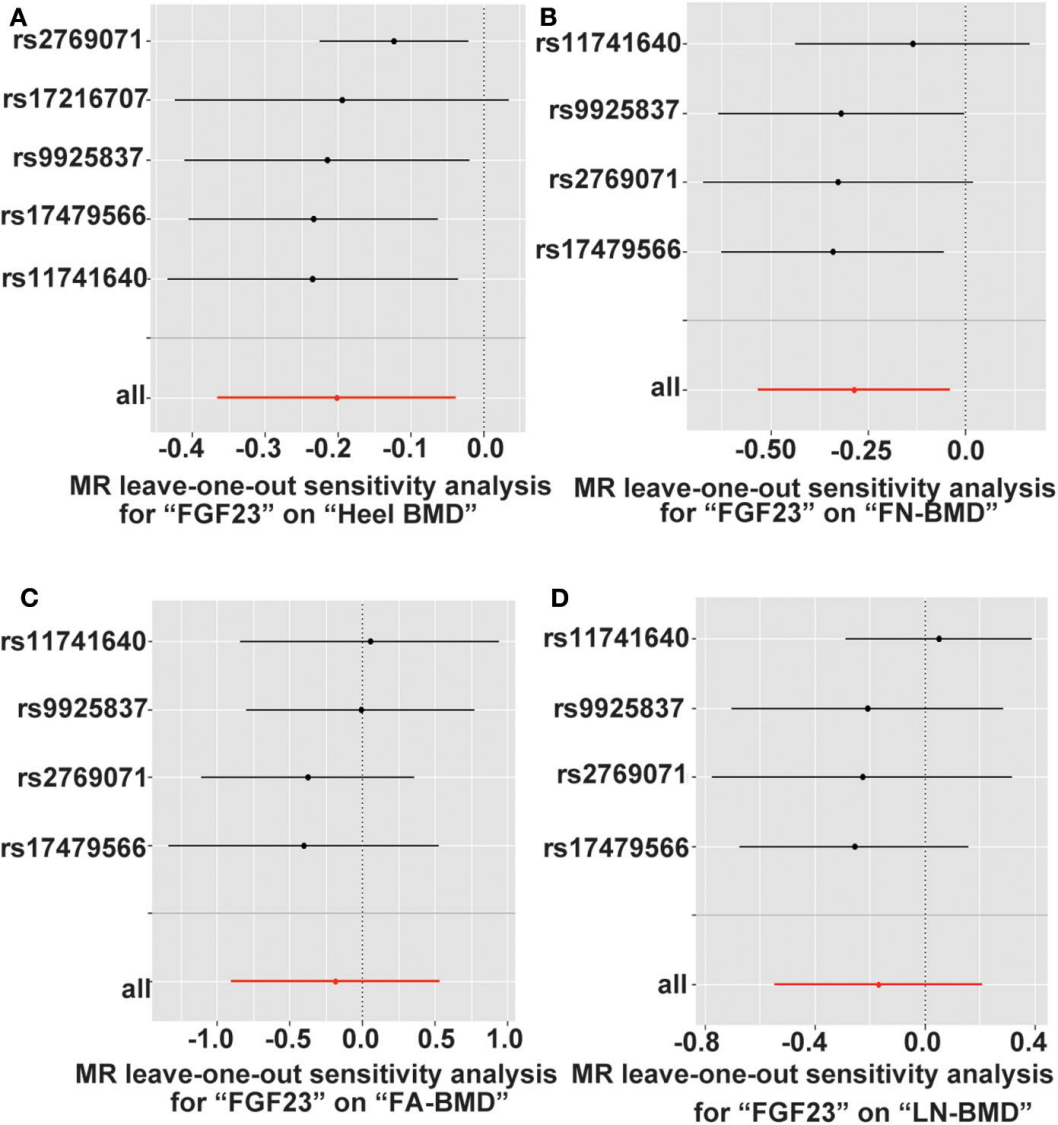
Leave-one-out sensitivity two-sample MR analysis based on the inverse variance weighted (IVW) model for FGF23 on **(A)** Heel-, **(B)** FN-, **(C)** FA-, and **(D)** LS-BMD. FN-BMD, femoral neck BMD; LS-BMD, lumbar spine BMD; FA-BMD, forearm BMD.

To assess the negative influences of FGF23 level on adult FN, LS, FA, and heel BMD and serum bone metabolic traits, we performed the leave-one-out sensitivity analysis using conventional IVW methods (Figure 3). The deletion of IV rs17216707 had modest influences on the causal relationship between FGF23 levels and the heel-BMD value. For FN-BMD, both 11741640 and rs9925837 have slight influence on causal inference results of FGF23 and FN-BMD (Figure 3). No heterogeneous SNPs that largely affected adult BMD and serum bone metabolic characteristics, as well as causal estimates in the leave-one-out analysis were observed (Figure S1).

## Discussion

The two-sample MR method has been widely accepted and used to assess the causal relationship between exposure and outcomes of diseases. In the current study, using five SNPs associated with FGF23 as main IVs, we found that the genetic susceptibility of FGF23 was significantly associated with the decrease in heel- and FN-BMD in adults. In contrast, no causal relationship was found between FGF23 levels and adult LS-, FA-BMD, and serum calcium, phosphorus, total vitamin D, creatinine, and magnesium levels. The results suggested that a higher FGF23 level was associated with reduced heel- and FN-BMD in adults, suggesting that FGF23 could be a novel therapeutic target for BMD in future.

This study was performed based on European individual summary statistics data from the EGFOS alliance and the MRC-IEU (24). The EGFOS alliance provided summary data for FA-BMD, FN-BMD, and LS-BMD, whereas MRC-IEU provided summary data for heel-BMD. FA-, FN-, LS-, and heel- BMD are reliable indicators for adult BMD level evaluation. Correlation of BMD value at different anatomic regions is lower, studies suggested evaluating BMD at multiple sites when investigating the relationship between exposure and adult BMD (25, 33). Here, we found that FGF23 demonstrated a causal relationship with sites of weight-bearing bones including the femoral neck and heel BMD, which indicated that FGF23 might have different influences on the BMD at different anatomic regions. Femoral neck and heel BMDs are commonly evaluated in epidemiological studies as sports and exercise are more susceptibility to weight-bearing bones, and studies have demonstrated that heel BMD is a valid measure of bone healthit, which is a predictor of future fracture and identifies increased fracture risk similar to hip BMD (34, 35). The bone mineral density of the femoral neck and lumbar spine of the elderly population is of great significance for the prediction of osteoporosis, measuring either the femoral neck or the lumbar spine BMD will correctly classify the majority of individuals as osteoporotic or not (36).

Previous studies have shown that FGF-23 is involved in the development of hypophosphatemia and bone diseases (37–44), these results suggest that FGF23 has an important role in bone metabolism. Mirza et al. found that when the serum FGF23 concentration was higher than 55.7 pg/mL, the risk of hip and non-vertebral fractures were significantly increased. They concluded that the risk of vertebral fractures and overall fracture risk in elderly men can be predicted by higher serum FGF-23 levels (45). The biological activity and physiological roles of FGF-23 are of great significance for mineral homeostasis (5). Shimada et al. reported that FGF-23 can induce hypophosphatemia 9 h following administration in a mice model, demonstrating the regulatory function of FGF-23 on serum phosphate (5). It has been suggested that impaired reabsorption of phosphate by FGF-23 is at least partly due to the decrease of NaPi-2a protein in the brush border membrane of proximal renal tubules, which is a key physiological molecule that determines the reabsorption of phosphate in the kidney. As an essential mineral in the human body, phosphate plays a vital role in several physiological processes, such as signal transduction, acid-base balance, energy production, and bone mineralization (46). However, our results did not support the relationship between FGF-23 and serum phosphate level. Serum phosphate level is affected by several factors, such as dietary phosphate intake, intestinal absorption, kidney reabsorption, and excretion (47). In addition to FGF23, phosphate metabolism is also affected by various regulators. This also includes the parathyroid hormone (PTH), calcitriol, and vitamin D (48). Therefore, it is reasonable that the relationship between FGF23 and serum phosphate concentration is difficult to establish using the current two-sample MR study.

FGF23 may affect BMD in adults by regulating 1α,25(OH)_2_D metabolism. Moreover, prior to reduction of the serum phosphate concentration, Shimada et al. found that FGF-23 can induce a significant decrease in serum 1α,25(OH)_2_D within 3 h after the injection (5). Recent studies on FGF-23 KO mice found an increased level of serum 1α,25(OH)_2_D, suggesting that FGF-23 is an indispensable factor in controlling serum 1α,25(OH)_2−_D levels (7). In addition, 1α,25(OH)_2_D has a negative feedback on FGF23 levels (49). Takashi Shimada et al. demonstrated that of 1α,25(OH)_2_D administration can induce elevated serum FGF-23 levels (5). However, the level of serum 1α,25(OH)_2_D is affected by several factors. For example, ultraviolet rays, eating habits, parathyroid hormone, calcium, and vitamin D conversion by the liver and kidneys (50). In recent years, widespread consumption of dietary supplements and vitamin D fortificated supplements or foods has made it difficult to obtain an accurate level of serum 1α,25(OH)_2_D. This made it more difficult to establish a correlation between FGF23 receptor gene polymorphism and serum 1α,25(OH)_2_D levels. Currently, we did not observe any significant association between the FGF23 and 25(OH)D level, the storage form of vitamin D, in the two-sample Mendelian randomization study. Whether FGF23 is causally associated with the 1α,25(OH)_2_D level needs to be addressed with more studies.

Magnesium is the fourth most abundant mineral (51), closely related to bone physiology (52). Magnesium is a divalent cation essential for bone and calcium metabolism. Normal PTH secretion requires sufficient plasma magnesium (52). Studies have shown that serum magnesium concentration is least sensitive to magnesium status, as only 1% of magnesium is in peripheral blood (53, 54). Hiroshi et al. reported an association between a low magnesium diet in mice and elevated serum FGF23 level, but whether FGF23 affects magnesium metabolism is still unknown due to a complex regulatory mechanism (55). Similarly, factors that affect serum calcium ion concentration are also extremely complex. Age, dietary habit, intestinal calcium absorption, and excretion, 1α,25(OH)_2_D, calcitonin, and parathyroid hormone can all affect serum calcium ion concentration (49, 52). Therefore, the null association between FGF23 and serum calcium concentration may be caused by various factors related to calcium absorption and metabolism, or the lower statistical power of the current Mendelian randomization study. In addition, blood calcium homeostasis is maintained in the included population. When serum calcium decreases, bone calcium is precipitated for compensation. However, further study is warranted to investigate the relationship between FGF23 and serum mineral levels to elucidate the molecular mechanism.

The advantages of this study are the large number of participants, including both men and women, and that the MR method can effectively control the confounding factors. To our knowledge, the current study is the first to evaluate the correlations between FGF23 levels and BMD with MR methods. Despite those and other advantages, our research inevitably has several limitations. Firstly, genetic variation by MR analysis is usually carried out by instrumentation. The causal link between FGF23 and 1α,25(OH)_2_D, calcium, and other metabolic characteristics may be masked due to the insufficient statistical capabilities of the study. These loci only explain 3% of FGF23 variation, thus, additional influential loci are necessary as IVs. Secondly, although the MR-Egger regression analysis shows that the main MR analysis results are not affected by pleiotropy effects, other confounding factors including population stratification and contact time, may have an effect. Lastly, the conclusions made in this study were primarily dependent on aggregated data of the GWAS conducted in a population of European ancestry. Therefore, these results need to be verified in populations with other ancestries. In conclusion, the current MR analysis demonstrated that an increase in plasma FGF23 concentration is associated with the reduction of the heel- and FN-BMD in adults, rather than lumbar spine BMD and forearm BMD. These findings indicate that FGF23 can be used as a biomarker to identify the risk of heel BMD in adults; however, more prospective cohort studies and large-scale community intervention investigations are required to confirm these results.

## Data Availability Statement

Publicly available datasets were analyzed in this study. This data can be found at: http://phewas.mrbase.org/.

## Author Contributions

HW and PC designed, researched, and revised the manuscript strictly. YW analyzed the data set and wrote the manuscript. All authors approved the final version to be published.

## Conflict of Interest

The authors declare that the research was conducted in the absence of any commercial or financial relationships that could be construed as a potential conflict of interest.

## Supplementary Material

Figure S1 Leave-one-out sensitivity analysis on the IVW model of 2-sample MR study for FGF23 on Ca **(A)**, Cr **(B)**, Mg **(C)**, VD **(D)**, P **(E)**, and OA **(F)**. Cr, serum creatinine; P, serum phosphorus; Ca, Calcium; Mg, Magnesium; OA, Osteoarthritis; VD, Vitamin D.
