# Supplementary figures and images for: Higher Fibroblast Growth Factor 23 Levels Are Causally Associated With Lower Bone Mineral Density of Heel and Femoral Neck: Evidence From Two-Sample Mendelian Randomization Analysis
Source: Front Public Health. 2020 Sep 2;8:467. doi: 10.3389/fpubh.2020.00467 (PMC7492544; doi:10.3389/fpubh.2020.00467)

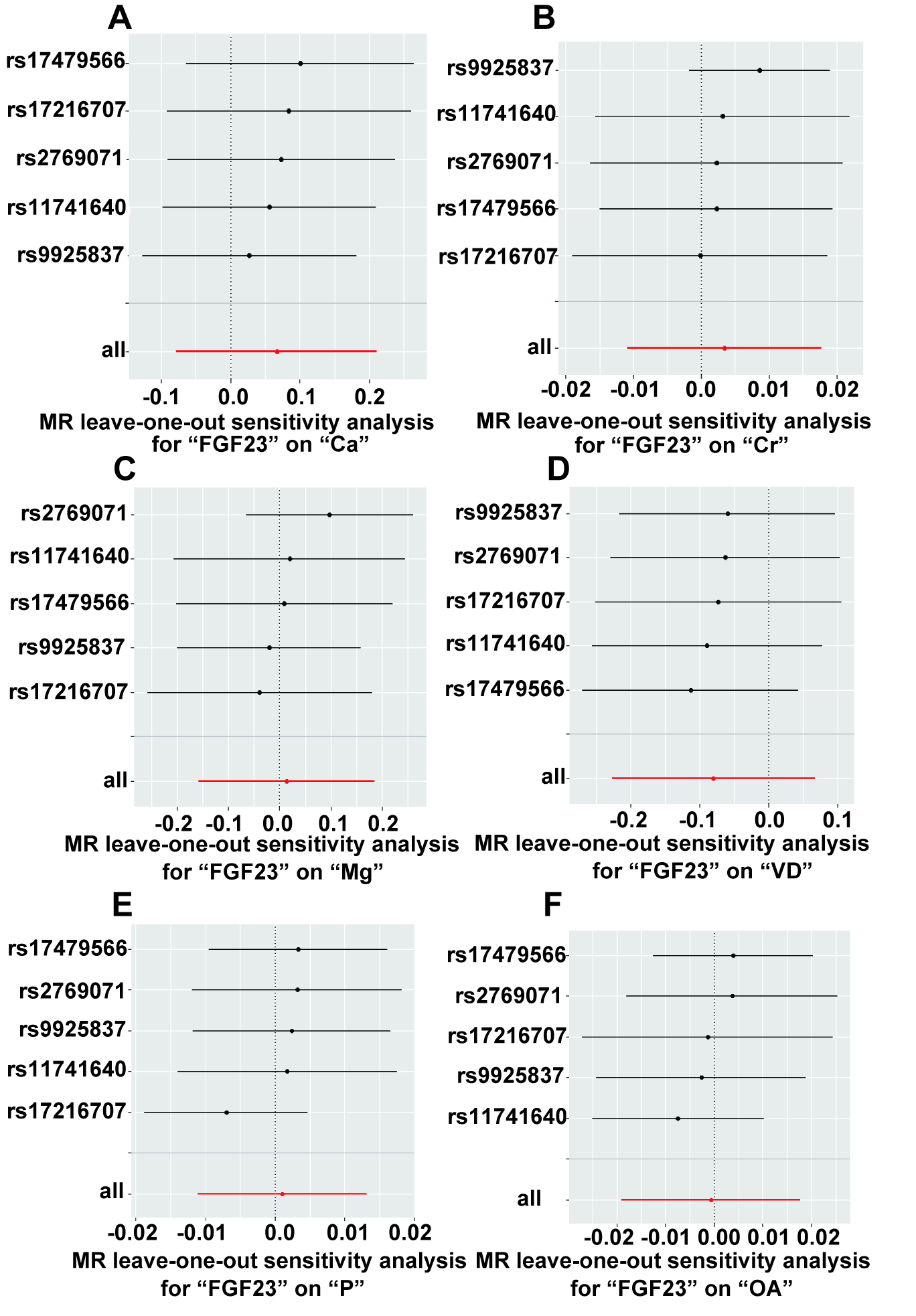

Supplement: Figure S1 — Leave-one-out sensitivity analysis on the IVW model of 2-sample MR study for FGF23 on Ca (A), Cr (B), Mg (C), VD (D), P (E), and OA (F). Cr, serum creatinine; P, serum phosphorus; Ca, Calcium; Mg, Magnesium; OA, Osteoarthritis; VD, Vitamin D. [file Image_1.TIF]
