# Supplementary material for: Higher Fibroblast Growth Factor 23 Levels Are Causally Associated With Lower Bone Mineral Density of Heel and Femoral Neck: Evidence From Two-Sample Mendelian Randomization Analysis
Source: Front Public Health. 2020 Sep 2;8:467. doi: 10.3389/fpubh.2020.00467 (PMC7492544; doi:10.3389/fpubh.2020.00467)
Supplement: Supplementary file 2 [file Table_1.doc]

**Supplementary Table 1**. Participant characteristics of genome-wide association studies on FGF23 and EGFOS.

| **Characteristics** | **GWAS on FGF23** | **EGFOS GWAS data** |  |
| --- | --- | --- | --- |
| **Participants, n** | ARIC, 8,594; CHS, 1,988; Indiana, 1,128; MESA, 2,163;MrOS GBG, 937; MrOS Malmo, 894; OPRA, 920. | 58,878 |  |
| **Female, n (%)** | ARIC, 4,632 (53.9); CHS, 1,234 (62.7); Indiana, 1,128 (100); MESA, 1,138 (52.7); MrOS GBG, 0 (0); MrOS Malmo, 0 (0); OPRA,920 (100) | 36,505 (62.0) |  |
| **Age,yr (SD)** | ARIC, 57.1 (5.7); CHS, 78.0 (4.4); Indiana, 36.4 (8.5); MESA, 62.6 (10.3); MrOS GBG, 72.3 (5.7); MrOS Malmo, 73.2 (5.8); OPRA,75.2 (0.1) | 62.3 (9.7) |  |
| **BMI, kg/m^2^** | ARIC, 27.3 (5.0); CHS, 26.6 (4.5); Indiana, 26.3 (6.0) ; MESA, 27.7 (5.1); MrOS GBG, 27.5 (3.7); MrOS Malmo, 27.5 (3.7); OPRA, 26.2 (4.1); | 26.8 (—) |  |
| **FGF23*** | pg/ml (intact): ARIC, 45.5 (17.3); Indiana, 39.4 (17.4); MESA, 41.9 (17.6); MrOS GBG, 46.2 (22.5); MrOS Malmo, 55.9 (27.7); pg/ml (C-terminal): CHS, 12.9 (21.3); OPRA, 8.3 (9.1) | — |  |
|  |
| **Ancestor** | European | European |  |

**Abbreviations:** ARIC, the Atherosclerosis Risk in Communities Study; Indiana, the Indiana Sisters Study; MrOS GBG, Osteoporotic Fractures in Men Study-Goteborg; MESA, the Multi-Ethnic Study of Atherosclerosis; MrOS Malmo; OPRA, the Osteoporosis Prospective Risk Assessment Study; CHS, the Cardiovascular Health Study. The FGF23 levels in the GWAS are serum FGF23 for MESA, ARIC, Indiana, MrOS and OPRA, but plasma in CHS. Data are mean (SD) or number (%), as appropriate. —, not available.

* The FGF23 levels in the GWAS are determined in serum for MESA, ARIC, Indiana, MrOS and OPRA, but plasma in CHS cohort.
